# Screening, isolation and characterization of biosurfactant producing *Bacillus subtilis* strain ANSKLAB03

**DOI:** 10.6026/97320630014304

**Published:** 2018-06-30

**Authors:** Anuraj Nayarisseri, Poonam Singh, Sanjeev Kumar Singh

**Affiliations:** 1 Computer Aided Drug Designing and Molecular Modeling Lab, Department of Bioinformatics, Alagappa University, Karaikudi-630 003, Tamil Nadu, India; 2 In silico Research Laboratory, Eminent Biosciences, Indore - 452 010, Madhya Pradesh, India; 3 Corrosion & Materials Protection Division-/C.S.I.R - Central Electrochemical Research Institute, Karaikudi 6300 06, India

**Keywords:** Biosurfuctant, bacteria, Bacillus subtilis, 16S rRNA, sequence

## Abstract

Biosurfactants are surface-active compounds produced by a wide range of microorganisms. They have both hydrophobic and hydrophilic domains and can decrease the surface tension and the interfacial tension of growth medium. Biosurfactants have different chemical structures like-lipopeptides, glycolipids, neutral lipids and fatty acids. They are biodegradable non-toxic biomolecules that show strong emulsification of hydrophobic compounds. They have the ability to form stable emulsions. The low water-solubility of these compounds restricts their availability to microorganisms. Surfactants secreted by microbes enhance the bioavailability of such hydrophobic compounds for bioremediation. Therefore, biosurfactant-enhanced solubility of pollutants has prospective applications in bioremediation. Biosurfactants are useful in a variety of industrial processes, and are also of vital importance to the microbes in adhesion, emulsification, and bioavailability, desorption and defense strategy. Therefore, it is of interest to identify biosurfuctantproducing strain of bacteria from brackish water. The microbial samples were isolated from the Chilika Lake, odisha, India and were tested for its biosurfactant property by various biochemical methods. 16S rRNA was sequenced using Sanger dideoxy sequencing method to characterize the biosurfuctant producing strain. The new Bacillus subtilis strain ANSKLAB03 isolated from 40 samples was deposited in GenBank with accession number KU523257.

## Background

Biosurfactants are the amphiphilic compounds with the ability to accumulate between fluid phases and are produced on microbial cell surfaces or can be secreted extracellularly [1]. The hydrophilic moiety of the biosurfactants can be a carbohydrate, an amino acid, a phosphate group, or alike compounds whereas the hydrophobic moiety is mostly the fatty acid carbon chain. This property helps reducing the interfacial and surface tensions, making them potential candidates for enhancing oil recovery [2]. A number of microorganisms have been stated to produce a number of classes of biosurfactants such as glycolipids, lipopeptides, phospholipids, neutral lipids or fatty acids and polymeric biosurfactants [3, 4, 5]. Few of the Pseudomonas sp. are found to produce a group of biosurfactants called rhamnolipids [6, 7, 8]. Cooper and Paddock in 1983 found several species of Torulopsis producing sophorolipids group of surfactants. There are lipopolysaccharides, such as emulsan, synthesized by Acinetobacter sp. [9] and lipoproteins or lipopeptides, such as surfactin and subtilisin, produced by Bacillus subtilis [10, 11]. Other effective biosurfactants are mycolates and corynomycolates which are produced by Rhodococcus sp., Corynebacteria sp., Mycobacteria sp. and Nocardia sp. [12, 13] and ornithinlipids, which are produced by Pseudomonas rubescens, Gluconobactercerinus, and Thiobacillus ferroxidans [14, 15]. Surfactin is one of the most effective lipopeptide biosurfactants produced by B. subtilis. It reduced the surface tension of water from 72 mN/m to 27 mN/m [16]. Moreover, surfactin can be used for improving the treatment of residual hydrocarbon from ship bilge waste [17]. Addition of a non-sterile biosurfactant obtained from B. subtilis O9 could enhance biodegradation of aliphatic hydrocarbons from 20.9% to 35.5% and of aromatic hydrocarbon from nil to 41% [18]. Prommachan (2002) reported that B. subtilis MUV4 produced the lipopeptide biosurfactant 0.8 g/L in Mckeen Medium with 2.5% glucose [19]. Biosurfactant can make hydrocarbon complexes more mobile with its potential use in oil recovery, pumping of crude oil and in bioremediation of crude oil contaminant. Ex situ MEOR studies of B. subtilis (DM-03 and DM-04) by using a sandpacked column showed that the two strains were effective in oil recovery from sand pores [20]. Due to their interesting properties such as lower toxicity, higher degree of biodegradability, higher foaming capacity and optimal activity at extreme conditions of temperatures, pH levels and salinity, they have been increasingly attracting the attention of the scientific and industrial community [21]. The application of bio-surfactant is reviewed elsewhere [22] [23]. Several factors affect production of bio-surfactants, such as the nature of carbon and nitrogen sources used, as well as the presence of phosphorus, iron, manganese and magnesium. In addition, other factors such as pH, temperature, agitation and operation mode are extremely important to quantity and quality of produced biosurfactant [24]. Therefore, it is of interest to identify, isolate and characterize new strains producing biosurfactants from marine sources.

## Methodology

### Sample Collection

The water samples were collected from Chilika Lake, which is largest brackish water lagoon in India with great genetic diversity. The sample was collected from oil-contaminated site of Chilika Lake in sterile 50ml tube. The sample was immediately stored at 4°C till usage to preserve the microbial consortium of water sample.

### Isolation of Microbial Consortium

The water samples collected from Chilka Lake were enriched using inoculating in sterile Mineral Salt Medium (MSM). 1 ml of sample was inoculated 100 ml of minimal salt medium containing (in g/L): 15g NaNO3, 1.1g KCl, 1.1g NaCl, 0.00028g FeSO4.7H2O, 3.4g KH2PO4, 4.4g K2HPO4, 0.5g MgSO4.7H2O, 0.5g yeast extract at 37°C in shaker incubator (100 rpm). After 24 hours of incubation, the samples were selected based on the colony morphology on nutrient agar. The selected isolates were screened for the production of biosurfactants using the following screening methods [25].

### Screening of Biosurfactant producing Isolates

Bacteria were grown aerobically in 500 ml Erlenmeyer flask with 100 ml of mineral salt medium containing (gl-1) 1.0 K2HPO4, 0.2 MgSO4.7H2O, 0.05 FeSO4.7H2O, 0.1 CaCl2.2H2O, 0.001 Na2MoO4.2H2O, 30 NaCl and crude oil (1.0%, w/v). Flasks containing sterilized mineral salt medium were inoculated with a loopful of bacterial culture grown in crude oil containing nutrient agar plates and the culture flasks were maintained in a shaker for 7 days at 200 rpm and 30°C. After 7 days of incubation, culture broth from each flask was centrifuged at 6000 rpm and 4°C for 15 minutes and the supernatant was filtered through 0.45μm pore size filter paper (Millipore). This cell free culture broth was used for drop collapse assay; oil spreading assay, emulsification assay and surface tension measurement and the bacterial cells were used for BATH assay. All the screening experiments were performed in triplicates (until otherwise mentioned) and the mean values were used as results [26].

### Oil spreading test

Oil spreading experiment was performed using the method described by Morikawa et al, 2000 [27]. In brief, 20 ml of distilled water was added to a plastic Petri dish followed by addition of 20 μl of crude oil to the surface of the water. 10 μl of cell free culture broth was then added to the oil surface. If biosurfactant is present in the cell free culture broth, the oil will be displaced with oil free clearing zone and diameter of this clearing zone indicates the surfactant activity, also called oil displacement activity. A negative control was maintained with distilled water (without surfactant), in which no oil displacement or clear zone was observed and Triton X-100 was used as the positive control.

### Drop collapse test

Screening of biosurfactant production was performed using the qualitative drop-collapse test described by Bodour and Maier in 1998 [28, 29]. Crude oil was used in this test. Two microlitres of oil was applied to the well regions delimited on the covers of 96- well micro plates and these were left to equilibrate for 24 hours. Five of the 48 hours culture, was transferred to the oil-coated well regions and drop size was observed after 1 min with the of a magnifying glass. The result was considered positive for biosurfactant production when the drop was flat and those cultures that gave rounded drops were scored as negative, indicative of the lack of biosurfactant production [30].

### Hydrocarbon overlay agar

ZMA plates were coated individually with 40 microlitre of kerosene, hexadecane, benzene and toluene. Pure bacterial isolates were spotted on these coated plates. Plates were incubated for 7-10 days at 28°C. Colony surrounded by an emulsified halo was considered positive for biosurfactant production.

### Bacterial adhesion to hydrocarbon (BATH) assay

Cell hydrophobicity was measured by bacterial adherence to hydrocarbons according to a method described by Rosenberg et al, 1980. The cell pellets were washed twice and suspended in a buffer salt solution (g/L, 16.9 K2HPO4 and 7.3 KH2PO4) and diluted using the same buffer solution to an optical density (OD) of ~ 0.5 at 610 nm. To the cell suspension (2 ml) in test tubes (10 ml volume with 10 x 100 mm dimension) 100 μl of crude oil was added and vortex-shaken for 3 min. After shaking, crude oil and aqueous phases were allowed to separate for 1 hour. OD of the aqueous phase was then measured at 610 nm in a spectrophotometer. From the OD values, percentage of cells attached to crude oil was calculated using the following formula:

% of bacterial cell adherence = (1-(OD_shaken with oil_/OD_original_)) x 100

Where: OD_shaken with oil_ - OD of the mixture containing cells and crude oil

OD_original_ - OD of the cell suspension in the buffer solution (before mixing with crude oil)

A few drops of 2-(4-iodophenyl)-3-(4-nitrophenyl)-5- phenyltetrazolium chloride (INT) solution was added to the above BATH assay solution and observed under a light microscope. The INT turned red if it was reduced inside the cells, indicating the viability of the cells adhered to the crude oil droplets [31].

### Calculation of Emulsification index (E_24_)

Several colonies of pure culture were suspended in test tubes containing 2 ml of mineral salt medium after 48 h of incubation; 2 ml petroleum was added to each tube. Then, the mixture was vortexed at high speed for 1 min and allowed to stand for 24 hours. The emulsion index (E24) [32].

Emulsification index=(Height of the emulsion layer/Total height) *100

### Surface tension analysis

Surface tension measurement of cell-free culture broth from each strain was determined in an atensiometer, using the du Nouy ring method [33]. Triton X-100 solution prepared at 1mg/ml concentration was used as a standard.

### Biochemical Characterization

Biochemical characterization of the microbial consortium was performed for the best three organisms. 24 hour activated culture was used to perform the tests. Cells were also inoculated on to selective Glutamate Starch Phenol red agar and were further incubated for 48 hours at room temperature. Further, KOH and Vancomycin tests were also performed.

### Optimization of Biosurfactant production by B. subtilis

#### Effect of pH

For optimization of pH, six pH values were selected, 5.0, 6.0, 7.0, 8.0, 9.0, and 10.0. MSM was prepared by adding 1% glucose as the sole carbon source and pH was adjusted by pH meter using 0.1N HCl and 0.1N NaOH solutions. After pH adjustment, the medium was sterilized at 121°C for 15 min. Activated culture of B. subtilis (1.8 x 104 CFU ml-1) was inoculated and incubated at 37°C for 7 days in orbital shaker at 150 rpm [34].

#### Effect of Temperature

For optimization of temperature, five temperature values were selected, 20°C, 30°C, 40°C, 50°C and 60°C. MSM was prepared by adding 1% glucose as the sole carbon source and pH was adjusted to 7.0 and sterilized at 121°C for 15 min. Activated culture of B. subtilis (1.8 x 104 CFU ml-1) was inoculated and incubated at different temperatures for 7 days in orbital shaker at 150 rpm [34].

#### Effect of Carbon

Eight carbon sources were taken, crude oil, coconut oil, diesel oil, sucrose, starch, glycerol, mannitol, and maltose. MSM was prepared with 1% of each carbon source and pH of the medium was adjusted to 7.0 and sterilized at 121°C for 15 min. Activated culture of B. subtilis (1.8 x 104CFU ml-1) was inoculated and incubated at 37°C for 7 days in orbital shaker at 150 rpm [34].

#### Effect of Nitrogen

Eight nitrogen sources were selected, ammonium nitrate, ammonium phosphate, ammonium sulfate, ammonium chloride, peptone, potassium nitrate, yeast extract, and urea. MSM was prepared with 1% sucrose carbon source and 1g/l concentration of each nitrogen source. pH was adjusted to 7.0 and sterilized at 121°C for 15 min. Activated culture of B. subtilis (1.8 x 104 CFU ml-1) was inoculated and incubated at 37°C for 7 days in orbital shaker at 150 rpm [34].

#### Effect of the Carbon and Nitrogen Concentration

The best optimized carbon and nitrogen sources were then optimized for best concentration required for maximum production. Carbon and nitrogen sources were added separately in the MSM at different concentrations: 1, 2, 3, 4, and 5%. pH of the medium was adjusted to 7.0 and sterilized at 121°C for 15 min. Activated culture of B. subtilis (1.8 x 104 CFU ml-1) was inoculated and incubated at 37°C for 7 days in orbital shaker at 150 rpm [34].

### Analysis for Optimization Conditions and Biosurfactant Extraction

At end of each optimization process, the bacterial cells were centrifuged for 20 min at 13,500g at 4°C and the supernatant were collected for emulsification activity. The optimal growth conditions were confirmed by emulsification activity and bacterial biomass in each parameter. Bacterial biomass was obtained by the process described elsewhere [35].

### Biosurfactant Extraction

The culture of Bacillus subtilis was inoculated in 100 ml of optimized medium incubated at 25°C for 7 days in a shaking incubator at 120 rpm. The cells were then removed by centrifugation at 5000 rpm, 4°C for 20 minutes. The supernatant was taken and the pH of the supernatant was adjusted to 2, using 1M H2SO4. Then equals volume of chloroform: methanol (2:1) was added. This mixture was shaken well for mixing and left overnight for evaporation. White colored sediment obtained is biosurfactant [36].

### Dry weight of biosurfactants

Sterile petriplate was taken and the weight of the plate was measured. Now the sediment was poured on the plates. They were placed on the hot air oven for drying at 100°C for 30 minutes. After drying, the plates were weighed [36]. The dry weight of the biosurfactants was calculated by the following formula:

Dry weight of biosurfactants = (Weight of the plate after drying - weight of the empty plate)

### DNA isolation

5 ml of overnight culture was centrifuged for 10 min at 10,000 rpm. 875βl of TE buffer was added to the cell pellet. 100βl of 10% SDS and 5βl of Proteinase K was added to the cells. The mixture was mixed well and incubated at 37°C for 1 hour. 1 ml of phenolchloroform mixture is added to the vial and incubated at RT for 5 min. It was then centrifuged at 10,000 rpm for 10 min. This process was repeated again and supernatant was collected. To the supernatant, 100βl of 5M-sodium acetate was added and mixed gently. 2 ml of isopropanol was added and mixed till DNA is precipitated. It is then centrifuged at 5000 rpm for 10 min. The supernatant was removed and 70% ethanol was added and centrifuged at 5000 rpm for 10 min. It is air dried and stored with TE buffer.

### 16S rRNA Sequencing

Amplified products were detached on 1% agarose gels in 1x TAE buffer at10 V mm-1 for 90 min and observed with a UV transilluminator and documented with GelDocXR software (Biorad). The amplification product was purified using Gene jet Gel Extraction PCR purification kit according to the manufacturer's instruction. The purified PCR product was sequenced by Sanger dideoxy sequencing technology. DNA Baser tool were used to assemble both the forward and reverse trace files obtained from the sequencing. Clean traces were observed in both the traces. The assembled sequences were further saved in FASTA format for further bioinformatics analysis.

### Elucidation of rRNA Secondary Structure

Precise secondary structures are vital for understanding ribosomes, which are to a great degree substantial and very intricate.RNA secondary structure with emblematic portrayals of base pairs, two fold helices, coils, loops, and single-strands, give systems to understanding three-dimensional (3D) structure, collapsing and capacity of RNA, and for sorting out, refining, and representing a wide assortment of data. One of the first steps to understanding the mechanism of action of an RNA is to determine its structure [37]. To understand RNA sequence mechanism structural must be known. There are number of software is available for secondary structure prediction based on energy minimization and dynamic programming. Here in our present study we focus on accurate re-determination of 2° structures, primarily of rRNAs using UNA fold software. UNAfold is web server amalgamation of two servers mfold & DINAMelt. The mfold software computes a collection of optimal and suboptimal foldings as well as a triangular shaped plot called an energy dot plot (EDP). The DINAMelt web server simulates the melting of one or two single-stranded nucleic acids in solution. The goal is to predict a melting temperature for a hybridized pair of nucleic acids and also entire equilibrium melting profiles as a function of temperature [38].

## Results and Discussion

### Isolation and biochemical characterization

There were three different organisms found from the oil contaminated water samples of Chilika Lake, which showed good growth results on the MSM plates. These were purified and were characterized on the basis of biochemical tests. These three organisms were found to be Bacillus sp. (4.41x108 CFU/ml), Staphylococcus sp. (3.94x108CFU/ml) and Escherichia coli (4.2x108 CFU/ml) (Table 1), using Bergey's manual as reference.

### Screening for biosurfactant production

#### BATH assay

The procedure was developed to estimate the cell hydrophobicity. The positive strains indicate the affinity of bacterial cells towards hydrophobic substrate (Table 2). Highest cell adherence was observed with Bacillus (94.23±0.71) and the least was observed with E. coli (60.15±1.42). Staphylococcus showed adherence of (84.77±0.56). Positive cell hydrophobicity was reported as an indication of biosurfactant production. Visualization of bacterial cells adhered to crude oil confirmed the affinity of cells towards crude oil droplets.

#### Hydrocarbon overlay agar plate

The HOA plate method is used to identify hydrocarbon-clastic bacteria. It shows the hydrocarbon degrading activity of the organisms. Quantitative assessment of bioemulsifiers (Table 2) showed that E. coli did not give any growth on toluene plated medium and gave 1.6 mm, 0.4 mm and 0.7 mm zone of clearance with kerosene, hexadecane and benzene plated medium, respectively. Staphylococcus showed growth with the entire hydrocarbons plated medium with 0.2 mm, 1.3 mm, 1.5 mm and 1.4 mm diameter of clearance zone with kerosene, hexadecane, benzene and toluene plated medium, respectively. Bacillus gave negative results with kerosene and benzene but gave good results with hexadecane and toluene with 2.9 mm and 3.4 mm clearance zone diameter, respectively.

#### Drop collapse assay

Drop collapse assay is a sensitive test, which can give result with a very small amount of surfactant. Some strains give positive results with BATH assay but give negative test for drop collapse which might be because some bacterial cells act as biosurfactant themselves (Hommel RK, 1994) and have high cell hydrophobicity, but do not produce extracellular biosurfactants [39]. Cell free culture broth is used for the test and 5 to 10βl of surfactant solution is required to conduct a duplicate measurement. Corroborating with oil spreading assay, E.Coli gave round shaped droplet indicative of negative result for drop collapse assay while the other two organisms showed positive results with a flat droplet. Staphylococcus drop collapsed in 1 min 56 sec and the Bacillus drop collapsed in 58 sec (Table 3). To further confirm the biosurfactant production, the cell free culture broths of the organisms were subjected to oil spreading and surface tension measurement experiments.

#### Oil spreading assay

Oil spreading assay results showed corroboration with the drop collapse assay results in the way that the organisms found positive with drop collapse assay were positive for oil spreading assay as well. Morikawa et al. explained that the oil displacement area is directly proportional to the surface-active compound in the solution. However, in this study only the qualitative study to check the presence of surfactant was carried out. The results (Table 3) state that E. coli gave negative test for oil spreading assay whereas Staphylococcus and Bacillus gave positive result with a clearance zone of 1.8 mm and 2.5 mm respectively.

#### Surface tension measurement

Surface tension measurement of cell free culture broth showed reduction in surface tension. There was a direct correlation found between drop collapse, oil spreading and surface tension assays. Strain slightly active in any one of these methods was active in other two methods. Similar direct correlation between drop collapse method and surface tension was reported using Bodour and Miller-Maier. E. coli gave negative result for the test whereas Staphylococcus and Bacillus gave positive results with 42mN/m and 38mN/m surface tension, respectively (Table 3).

#### Emulsification index (E24)

Emulsification assay is an indirect method used to screen biosurfactant production. It was presumed that if the cell free culture broth contains biosurfactant then it would emulsify the hydrocarbons present. Here, crude oil was used as the hydrophobic substrate. The E24 of all three organisms are noted in Table 3. The values are meaning of three readings, emulsification index > 30% is indicated in bold to show high activity. Maximum emulsification was observed with Bacillus (87%) and minimum with E. coli (15%).

#### Optimization of Biosurfactant production

**Figure 4:**
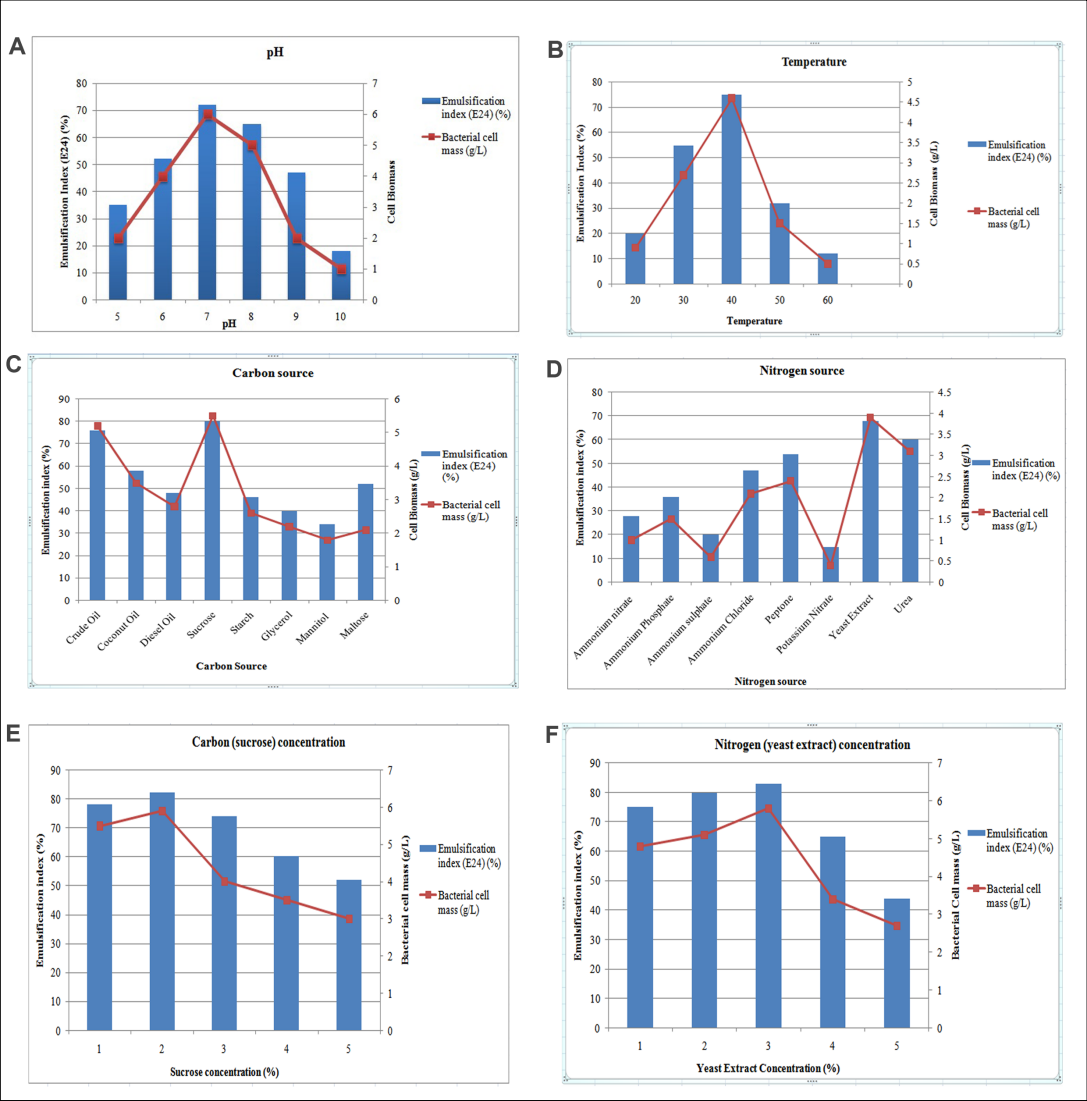
(a) Cell Biomass and emulsification index at different pH; (b) Cell Biomass and emulsification index at different temperatures; (c) Cell Biomass and emulsification index for different carbon sources; (d) Cell Biomass and emulsification index for different nitrogen sources; (e) Cell Biomass and emulsification index for different concentrations (%) of best carbon source (Sucrose); (f) Cell Biomass and emulsification index for different concentrations (%) of best nitrogen source (Yeast Extract).

The optimum pH was confirmed as 7.0 (E24: 72%), subsequently pH 8.0 showed a substantial effect (Figure 4a). Similarly, the optimum temperature was confirmed as 40°C (E24: 75%) (Figure 4b). The organism is mesophilic, which indicates it exhibits effective production level at moderate temperature (30°C-40°C). Among the carbon sources, sucrose was found the most favorable (E24: 80%) followed by crude oil (E24: 76%) (Figure 4c). Similarly among the eight nitrogen sources, yeast extract showed the highest E24 value (68%)followed by Urea (E24: 60%) (Figure 4d). All the optimized conditions were taken to prepare the production medium. Among the given 1-5% of the carbon and nitrogen sources, 2% of the sucrose (E24: 82%) (Figure 4e) and 3% of the yeast extract (E24: 83%) (Figure 4f) were optimal.

#### Extraction of biosurfactants and dry weight

The culture inoculated in mineral salt medium with oil was centrifuged and the supernatant was taken mixed with Chloroform: methanol. White sediment was retained was placed on an empty petri plate and was measured and recorded in Table 4. Maximum amount of biosurfactant produced by Bacillus was 0.324 g per 100 mL of medium.

#### Genotypic Characterization and sequencing

**Figure 5:**
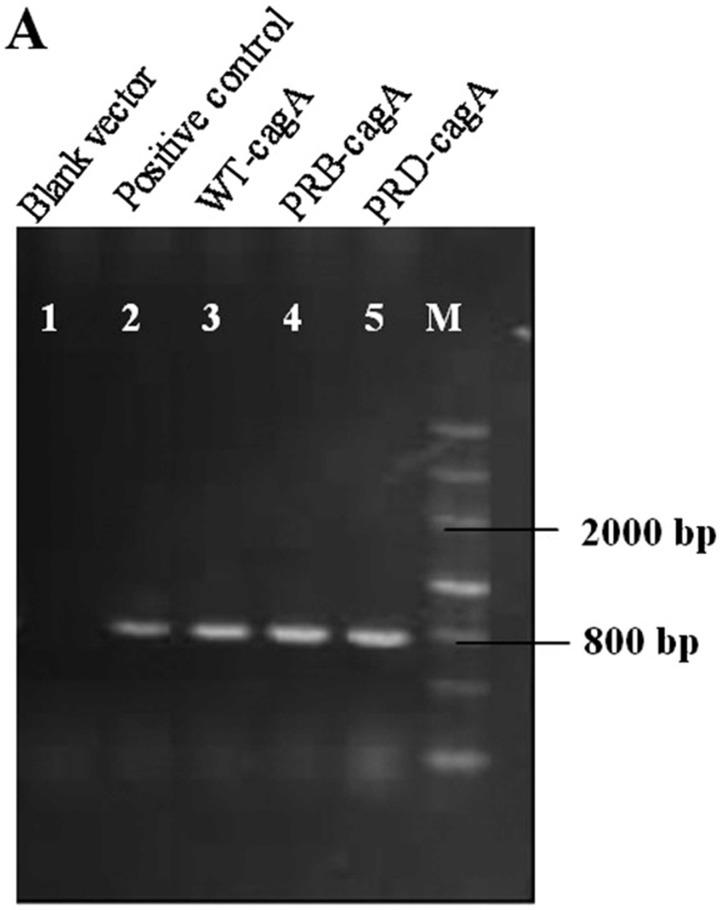
16S rRNA PCR product on 2% agarose gel (Lane M: Marker, Lane 1: blank, Lane 2-5: PCR Products).

Figure 5 shows amplified product of 16S rRNA gene electrophoreses on 2% agarose gel. The amplified product yielded a sequence file, further sequenced through automated Sanger dideoxy sequencing process. DNA Baser sequence assembler v.4.2.0, a sequence analysis algorithm was used to assemble both the forward and reverse ABI sequence trace files.

The sequence thus obtained from Sanger sequencing were used for pairwise local alignment against Genbank16S ribosomal RNA sequence database (Bacteria and Archaea) database using BLASTN 2.8.0 [40, 41, 42]. The HSPs obtained from Blast results found to have less similarity with available Bacillus species. The results drawn from sequence interpretation of the 16S rRNA gene of these isolates were found to be a novel strain of Bacillus subtilis sp., which were named Bacillus subtilis strain ANSKLAB03, and the sequence of the isolate was deposited in GenBank with accession number 'KU523257'.

#### 16S rRNA Phylogenetic Construction

**Figure 6:**
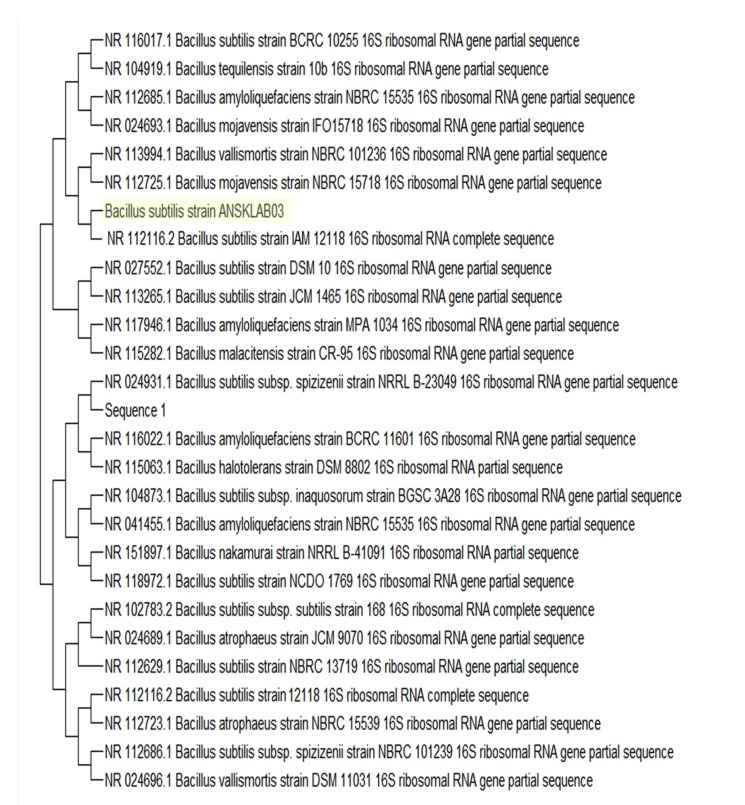
Phylogenetic affiliation of Bacillus subtilis strain ANSKLAB03 against other species of Bacillus subtilis

Phylogenetic construction of named Bacillus subtilis strain ANSKLAB03 against other species of named Bacillus subtilis strains is shown in Figure 6. The dataset of Bacillus subtilis strain ANSKLAB03 consisted of 957 bp, which were completely parsimony informative. Further, the matrix was manually aligned and missing data had no effect on the topology. Neighbor-joining method was used to calculate the evolutionary distances and construct phylogenetic tree which had optimal tree with the sum of branch length (SBL) = 1.25753874. The bootstrap test (1000replicates) is shown next to the branches. Bootstrapped phenograms were produced for all the trees using consensus procedure [43, 44, 45]. Trees were treated as sun rooted, the 'out group designation' option was included to polarize the character states. A comparative study of phylogenetic trees was performed by maximum-likelihood, UPGMA methods [42] and by maximum parsimony methods [45], which resulted in similar topologies of the strain Figure 6.

#### Elucidation of rRNA Secondary Structure

RNA structure thermodynamics has been successfully studied by methods like absorbance melting curves [46] and micro calorimetry, which includes isothermal titration calorimetry and differential scanning calorimetry [47]. The three dimensional structure of RNA assumes to have astounding number of shapes which proved to be a daunting task to predict the native structure in the equilibrium. In addition, there are numerous distinct motifs in RNA structure and the number of probable sequence combinations for most motifs are huge. However, in principle it is possible to predict the stable RNA structure by tenets of thermodynamics. The chief objective of thermodynamic principles is to provide a foundation to efficiently predict the structure from sequence. Given the present technology, however it is unlikely to establish thermodynamic parameters for all given possible helices with Watson-Crick base pairs. Therefore efficient and robust models have been developed to predict the thermodynamics of helix formation from limited set of measurements. Some of the prominent attempts include neighbor model in predicting stabilities of RNA duplexes with only Watson-Crick pairs by algorithms proposed by Borer et al. [42]. Also, Gray et al. [43] has offered an alternate exploration for thermodynamic properties of duplex formation, which includes determination of spectroscopic properties of RNA combined with thermodynamic properties. Yet in another approach, phylogenetic information and thermodynamic principles have been integrated for near prediction of RNA structure [42].

**Figure 7:**
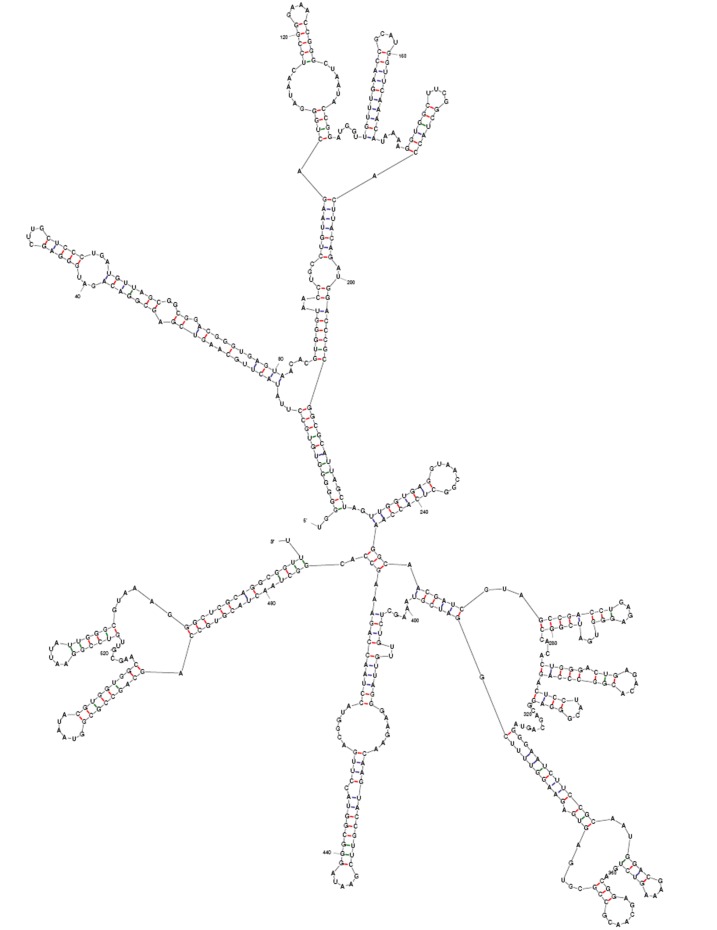
RNA secondary structure of Bacillus subtilis strain ANSKLAB03

The secondary structure showed helical regions, which bind with S1eS27 proteins, interior loops, hairpin loops, bulge loops, and multi-branched loops to bind 23S rRNA. The free energy (ΔG) of the secondary structures of Bacillus subtilis strain ANSKLAB03 were calculated to be -236.20kcal/mol as elucidated using UNAFOLD (Figure 6). The thermodynamic result from the each base of the dataset shows the average energy of external closing pair helix, stack, multi-loop, bulge loop, hairpin loop respectively to be ΔG = -1.90, ΔG = -5.10, ΔG = -2.40, ΔG = -6.70, ΔG = -9.20 and closing pair and interior loop of ΔG -4.50 kcal/ mol. Further, we used RNA fold server [42] for predicting the overall entropy of the of the rRNA structure of Bacillus subtilis strain ANSKLAB03 (Figure 7).

## Conclusion

Known bio-surfactants producing strains are from terrestrial origin. However, reports on marine biosurfactant molecules are limited. We report the isolation of three organisms from marine water sample that produce biosurfactant. E.Coli was not found producing extracellular biosurfactant, but acted as a biosurfactant itself [48]. Surfactin, a lipoheptapeptide produced by Bacillus subtilis, is one of the most effective biosurfactants known; it can reduce the surface tension (ST) of water up to 27 mN/m, with critical micelle concentrations (mmc) as low as 0.01 g/l, and shows a high emulsifying activity; furthermore, it exhibits antimicrobial, antiviral, and anti-tumor activities [49]. S. aureusis also found as a potential producer [50]. The surface tension of Bacillus was found to be the lowest (38 mN/m) indicating its powerful surface tension-reducing property. The potential to reduce surface tension depends largely on the molecular structure of the biosurfactant produced. The strain was optimized for its production of biosurfactants and best results were obtained with sucrose (2%) and yeast extract (3%) in the medium at 7 pH and 40°C temperature. These optimized conditions were then used to check the dry weight of biosurfactant produced by the organism. The organism, Bacillus subtilis strain ANSKLAB03 produced 0.324 g of biosurfactant in 100mL of medium. The described results of phenotypic disparities and phylogenetic distinctiveness suggest that Bacillus subtilis strain ANSKLAB03 is a novel strain of biosurfuctant producing bacteria. The free energy (ΔG) of predicted RNA structure of Bacillus subtilis strain ANSKLAB03 is -236.20 kcal/mol and is thermodynamically stable.

## Conflict of Interest

The authors confirm that this article content has no conflict of interest.

## Figures and Tables

**Table 1:** Biochemical characteristics of the microorganisms.

Biochemical characterization				
S.No.	Parameters	Microorganisms		
		E.Coli	Bacillus	Staphylococcus
1	Colonial Characters	Large, thick, greyish white, moist, smooth, opaque colonies.	Large, Circular, Uneven, Slightly Raised, Opaque, Smooth, Off-white colonies	Small, Round, Even, Flat, Transluscent, Smooth, Golden Yellow colonies
2	Pigmentation	No pigmentation	off - white Pigmentation	Yellow Pigmentation
3	Microscopic characters	Rod shaped	Rod shaped	Circular, cocci
4	Gram's Staining	Gram Negative	Gram Positive	Gram Positive
5	Glucose fermentation	Positive	Positive	Positive
6	Lactose Fermentation	Positive	Negative	Positive
7	Sucrose Fermentation	Positive	Positive	Positive
8	Mac Conkey agar	Pink colored colonies	No growth	No growth
9	Starch Hydrolysis	Negative	Positive	Negative
10	Lipid Hydrolysis	Negative	Positive	Positive
11	Casein Hydrolysis	Negative	Positive	Negative
12	Gelatin Hydrolysis	Negative	Positive	Negative
13	Indole Production	Positive	Negative	Negative
14	Methyl Red test	Positive	Positive	Negative
15	VogesProskeur	Negative	Positive	Negative
16	Citrate Utilization	Negative	Positive	Positive
17	Nitrate Reduction	Positive	Positive	Positive
18	Motility	Positive	Positive	Positive
19	Catalase Activity	Positive	Positive	Positive
20	H2S Production	Negative	Negative	Negative
21	Gas Production from Glucose	Positive	Positive	Positive
22	Urease Activity	Negative	Negative	Positive
23	Oxidase Activity	Negative	Negative	Negative
24	Spores	Negative	Positive	Negative
25	KOH gelling test	Positive	Negative	Negative
26	Vancomycin test	Negative	Positive (sensitive)	Positive (sensitive)

**Table 2:** BATH assay and Hydrocarbon overlay agar plate.

Microorganism	BATH assay^a^	Hydrocarbon overlay agar plate^b^			
Kerosene	Hexadecane	Benzene	Toluene
E.Coli	++	++	+	+	Nil
Staphylococcus	++	+	++	++	++
Bacillus	+++	Nil	+++	Nil	+++
BATH assay^a^: '+++' - cell adhesion > 90%, '++' - 60 to 89% cell adhesion, '+' - 40 to 59% cell adhesion; Hydrocarbon overlay agar plate^b^:'+' - clearance zone of 0.1-1 mm, '++' - clearance zone of 1.1 to 2 mm, '+++' - clearance zone of 2.1 to 3.5 mm.					

**Table 3:** Oil spreading assay, Drop collapse assay, Emulsification assay and Surface tension measurement

Microorganism	Drop collapse assay^b^	Oil spreading assay^a^	Surface tension measurement^d^	Emulsification index^c^
E.Coli	Nil	Nil	Nil	15%
Staphylococcus	++	++	++	58%
Bacillus	+++	++	+++	87%
Oil spreading assay^a^: '+' - oil spreading with a clear zone of 0.5-1.5 mm, '++' - oil spreading with a clear zone of 1.6 to 2.5 mm, '+++' - oil spreading with a clear zone of 2.6 to 3.5 mm; Drop collapse assay^b^: '+++'- drop collapse within 1 minute, '++'- drop collapse after 1minute and '+' - drop collapse after 3 minutes of biosurfactant addition; Emulsification assay^c^: values are mean of three readings, emulsification index >30% is indicated in bold to show high activity; Surface tension^d^: '+++'- surface tension <40 mN/m, '++'- surface tension 40 to 50mN/m, '+'- surface tension 51 to 70mN/m.				

**Table 4:** Dry weight of biosurfactant produced by the organisms.

Microorganism	Empty plate weight (g)	Biosurfactant containing plate weight (g)	Dry weight of biosurfactant (g)
Bacillus	47.254	47.578	0.324

**Figure 1:**
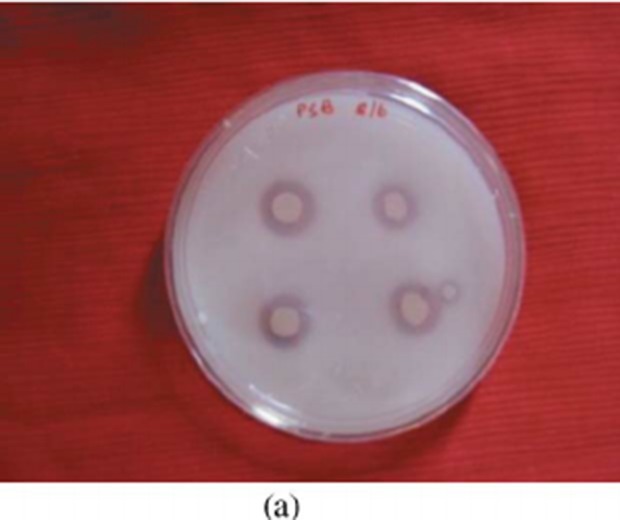
Hydrocarbon overlay agar test.

**Figure 2:**
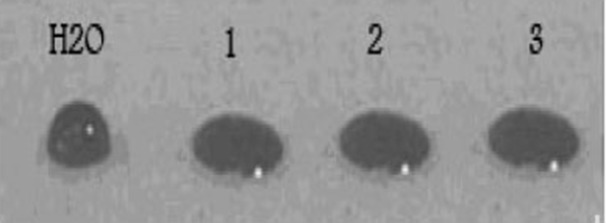
Drop collapse assay

**Figure 3:**
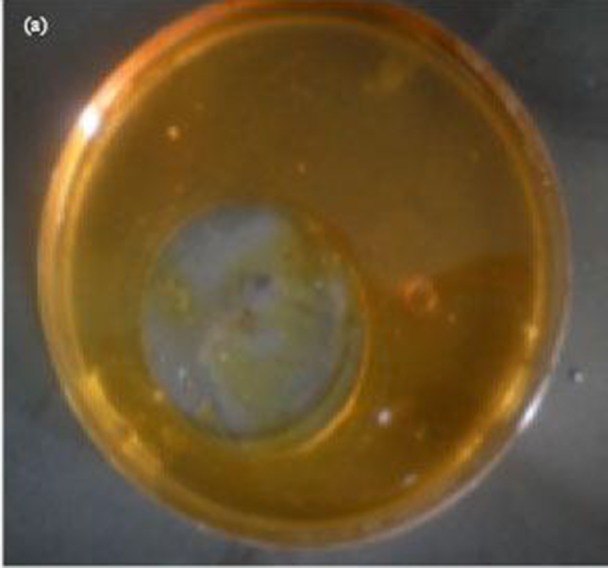
Oil spreading assay.
